# Supplementary material for: Pocket warming of bupivacaine with fentanyl to shorten onset of labor epidural analgesia: A double-blind randomized controlled clinical trial
Source: PLoS One. 2024 Sep 4;19(9):e0309515. doi: 10.1371/journal.pone.0309515 (PMC11373790; doi:10.1371/journal.pone.0309515)
Supplement: S1 File — (PDF) [file pone.0309515.s003.pdf]

Research Protocol

i. Study Title

Pocket Warming of Epidural Medications to Shorten Onset of Labor Analgesia

ii. Principal Investigator

Ling-Qun Hu, M.D.

I. Background and Rationale

A recent report by Sviggum et al.[1] described the beneficial effect of warming epidural medications in order to hasten the onset of labor analgesia. Epidural 0.125% bupivacaine with fentanyl 2mcg/mL at 37 °C resulted in a mean analgesic onset of 9.2 minutes as compared to a mean onset of 16 minutes for the same medication at room temperature.[1] Provision of neuraxial labor analgesia in a timely manner is important to many parturients,[2] and thus techniques to shorten analgesic onset are relevant to daily clinical practice. Combined-spinal epidural (CSE) techniques are commonly utilized to improve the onset [3] and reliability [3,4] of labor analgesia relative to epidural techniques, though CSEs have potential drawbacks. It is not always possible to administer a spinal dose despite successful loss-of resistance with CSE techniques.[5] CSEs have been associated with greater incidence of non-reassuring fetal heart tones (FHT), uterine hyperactivity, maternal pruritis, and greater incidence of neurologic sequelae compared to epidural analgesia.[6-9] Further, it has been reported that CSE labor analgesia is more likely to result in prolonged FHT decelerations if there is FHT abnormalities prior to the neuraxial procedure.[10] Given these potential limitations of CSE techniques for labor analgesia, epidural medication warming may represent an effective alternative.

Previous studies in obstetric [11] and non-obstetric [12,13] patients undergoing surgery have demonstrated a more rapid onset of sensory blockade in patients receiving body temperature (37 °C) epidural medication compared to room temperature medication. Though other reports have found no significant difference in the onset of sensory blockade when comparing body temperature to room temperature epidural medications.[14,15] Warmed local anesthetic solutions have also been shown to shorten the onset of peripheral nerve blockade with ropivacaine.[16] To our knowledge, the report by Sviggum et al. is the first to examine the effect of warmed epidural medications for patients receiving epidural labor analgesia.[1] The degree of local anesthetic ionization determines the onset time, and warming local anesthetics have been associated with a lower dissociation constant (pKa)[15] and increased pH,[13] thus causing more of the local anesthetic to exist in the unionized form that traverses the cell membrane before binding to the receptor site on the inside of the sodium channel.

Some of our prior colleagues have suggested that the pharmacy-prepared, sealed and labeled 10mL epidural medication syringes (0.125% bupivacaine with 2mcg/mL of fentanyl) should be kept in the front chest pocket of the surgical scrubs. In their experience, the close proximity to the body has a warming effect on the epidural medication and ultimately leads to quicker onset of pain relief once administered. Pocket warming does not warm the medications to the same extent as an incubator, but is certainly less expensive and is readily available to every obstetric anesthesia practice. For the purposes of this investigation, we will place the sterile epidural medication syringes within a clean plastic bag prior to placing it in the pocket in order to maintain cleanliness.

We have previously measured the temperature of five 10mL syringes of normal saline at room temperature and again after being placed in a clean plastic bag within the front chest pocket of five different anesthesia practitioners.[17] Using an STS-400 temperature sensor (Smiths Medical, Rockland, MA, USA) we measured the temperature of the test syringe contents. At baseline the average was 21.7 °C (range 21.5-21.9 °C), and increased to an average of 29.7 °C (range 29.1 – 30.2 °C) after 1.5 hours of pocket warming.[17] It has not been studied and is unknown if this degree of warming would be effective in enhancing the onset of labor analgesia, but such information is valuable given that an approximate 30 °C temperature may be accomplished by simple pocket warming and is within bupivacaine manufacturer recommended storage temperatures of 15-30 °C.[18]

We also assessed the potential for epidural medication cooling by measuring the temperature of one of our 10mL saline syringes for twenty minutes after removal from the warm pocket environment. The initial temperature of 30.0 °C had cooled to 27.0 °C by five minutes, 24.7 °C by ten minutes, 22.9 °C by fifteen minutes, and had returned to baseline room temperature by twenty minutes.[17] The time between medication removal and dosing is important to consider given that significant cooling may occur and negate any potential benefits of warming the medication. This cooling effect makes use of a centralized warmer less promising, as it could take 10-20 minutes to position the patient and complete placement of the epidural catheter prior to dosing the medication. The use of a bedside incubator[1] or a pocket warming technique[17] would be useful in this regard, because the medication could be administered immediately after removal from the warm environment.

As mentioned, local anesthetics warmed to body temperature (37 °C) have been shown to shorten the onset of sensory blockade.[1, 11-13, 16] We hypothesize that a lesser degree of warming, such as the 29.7 °C achieved by pocket warming,[17] would also be beneficial in enhancing onset of labor analgesia relative to room temperature medication. Pocket warming of medications has the additional benefits of being immediately available to every practitioner, requires no additional costs or equipment, and is within bupivacaine manufacturer recommended storage temperatures of 15-30 °C.[18]

II. Objectives

In comparison to labor analgesia initiation with room temperature local anesthetic medication, we hypothesize that local anesthetic with at least 1 hour of pocket warming will result in a more rapid onset of labor analgesia and will not result in any increase in adverse effects such as fever, shivering, hypotension, nausea or vomiting.

The warming of local anesthetic solutions may be effective in providing a more rapid onset due to alterations in the physiochemical properties of the drug. The degree of local anesthetic ionization determines the onset time, and warming local anesthetics have been associated with a lower dissociation constant (pKa)[15] and increased pH,[13] thus causing more of the local anesthetic to exist in the unionized form that traverses the cell membrane before binding to the inside of the sodium channel. Specific Aims:

1) To record the following information in laboring parturients receiving initiation of labor analgesia with room temperature epidural medication (0.125% Bupivacaine with Fentanyl 2mcg/ml – 10mL) vs. “pocket warmed” epidural medication (0.125% Bupivacaine with Fentanyl 2mcg/ml – 10mL) stored within a clean plastic bag in the front pocket of an anesthesia provider for a minimum of 1 hour:
    a. Patient characteristics
        i. Age
        ii. height (cm)
        iii. Current weight (kg)
        iv. Body Mass Index (kg/m^2) (BMI)
        v. Race
        vi. Gravidity
        vii. Parity
        viii. Gestational age
        ix. History of labor epidural in prior pregnancy
        x. Cervical dilation at initiation of labor analgesia (cm)
        xi. initial verbal rating scale (VRS) pain score (1-10)
        xii. Baseline patient temperature (°C)

xiii. Smoking status

b. Temperature (°C) of 10mL syringe of normal saline that is stored with the epidural medication syringe; note that directly measuring the temperature of the epidural medications to be administered to the patient is not advisable given sterility of medications would not be maintained, thus we intend to measure the temperature of a normal saline syringe of the same volume and stored in the same environment using an STS-400 temperature sensor (Smiths Medical, Rockland, MA, USA).

c. Analgesia onset time (minutes) – defined as the time from dosing epidural medication to achieving a VRS $\leq$ 3

   i. VRS pain scores will be assessed and recorded immediately after completion of the initial 10mL epidural bolus (time 0), and at 5, 10, 15, 20, 30 and 60 minutes

   ii. The time will also be recorded at the end of the first uterine contraction with VRS $\leq$3.

d. Oral temperature

   i. will be assessed and recorded immediately after completion of the initial 10mL epidural bolus (time 0) and again at 15, 30, 60 minutes. Note that fever will be defined as temperature >38 °C

   ii. the presence and magnitude of fever anytime during labor will also be recorded

e. Patient satisfaction, as assessed by a 1-100 rating scale

   i. Will be assessed on postpartum day # 1 during routine follow-up visit.

f. The following will be assessed and recorded immediately after completion of the initial 10mL epidural bolus (time 0) and again at 5, 10, 15, 20, 30 and 60 minutes:

   i. Shivering (0=none, 1=intermittent mild shivering, 2=continuous intense shivering)

   ii. Nausea (0=none, 1=mild, 2=moderate to severe (subjective assessment based on patient description))

   iii. Vomiting (yes/no)

   iv. Incidence of Hypotension – defined as systolic BP <90mm Hg, a decrease in systolic BP $\geq$ 20% from the pre-procedure systolic BP, or a symptomatic decrease from the pre-procedure baseline requiring treatment with vasopressors and/or IV fluid bolus

      1. Treatment with vasopressors due to hypotension will also be recorded

g. Breakthrough pain:

   i. Required rescue epidural bolus (yes/no)

   ii. Time to first manual epidural rescue bolus (minutes)

   iii. Total number of manual anesthetic interventions to treat inadequate labor analgesia

h. Type of delivery (cesarean, spontaneous vaginal or instrumental vaginal delivery)

The method of delivery will not influence assessment of pain in the first hour after epidural placement. The pain after vaginal delivery or after potential cesarean delivery will not be measured, thus the mode of delivery will not affect pain assessment.

i.

III. Methods and Materials

A. Research Design

For the purposes of attempting to safely achieve a more rapid onset of labor analgesia and improve patient satisfaction, we propose to conduct a prospective, randomized controlled trial to compare the initiation of labor with room temperature epidural medication (pharmacy-prepared 0.125% Bupivacaine with Fentanyl 2mcg/ml – 10mL) to the same epidural medication that has been pocket warmed for a minimum of 1 hour. The primary outcome is to assess whether the pocket warmed medication significantly shortens analgesia onset. We also intend to investigate whether there are significant differences in patient satisfaction, management of breakthrough pain, type of delivery, or adverse patient events such as nausea, vomiting, shivering, hypotension and fever. We will record baseline patient characteristics including age, height, weight, cervical dilation at time of labor analgesia, initial verbal rating scale (VRS) pain score (1-10), and baseline patient temperature (°C).

B. Subject Selection

1) Inclusion Criteria: Subjects who meet the following criteria will be included in the study.

    a. Women with a single vertex presentation fetus at term (38-42 weeks) with intact fetal membranes or membrane rupture ≤6 hours previously, who request to have an epidural for labor analgesia and provide written consent for the study.

2) Exclusion Criteria: Subjects meeting any one of the following conditions will be excluded from the study.

    a. Patients being treated/managed for chronic pain
    b. Allergies or significant adverse reactions to local anesthetic or opioid medications
    c. Contraindication to labor epidural placement
    d. Patients with history of spine abnormalities or spine surgery
    e. Clinical signs or symptoms of infection
    f. Baseline temperature > 37.6 °C
    g. Non-English speaking
    h. Prisoners
    i. Age less than 18 years old

C. Methods

1) Subject recruitment will be performed by one of the study investigators. Subjects will only be accrued if they meet all inclusion criteria, have no excluding factors and provide signed consent.

2) Patient characteristics will be recorded:
    a. age, height, weight, BMI, race, smoking status, gravidity, parity, gestational age, cervical dilation at time of labor analgesia, initial verbal rating scale (VRS) pain score (1-10), and baseline patient temperature (°C).

3) Epidural Placement:
    a. Patients selected and consented to participate in this investigation will receive placement of a labor epidural by an experienced anesthesia provider.
    b. Sterile prepping with ChloraPrep® and sterile draping will occur in the usual fashion. Cap, mask and sterile gloves will be worn by the anesthesia providers.
    c. Skin and subcutaneous tissue local anesthetic infiltration will be performed with 1% lidocaine prior to attempts at labor epidural placement at L3-L4 or L4-L5 interspace.
    d. A loss-of-resistance to saline (< 2 mL) epidural technique will be performed with a 17G or 18G Tuohy needle, and a BBraun Perifix® FX Springwound or BBraun Perifix® ONE epidural catheter (depending on anesthesia provider preference) will be placed 4-6 cm in the epidural space.
    e. Epidural catheter aspiration will be performed to ensure that intrathecal or intravascular placement of the catheter did not occur.

f. Epidural catheter test dose with 1.5% lidocaine with 1:200,000 epinephrine 3mL will be completed to ensure that no signs or symptoms of inadvertent intravascular or intrathecal catheter placement are present.

4) Initiation of labor analgesia: After randomization performed by the unblinded research staff, subjects will be randomly assigned to one of two groups (room temperature or pocked warmed). The blinding process will be maintained by ensuring blinded research staff will not be present during epidural placement. Therefore, subjects, clinicians, and blinded research personnel will be blinded throughout the study. Only the anesthesia care providers and/or unblinded research staff will know to which allocation the patient is randomized to.

a. After consent, patients will be randomized and allocated into one of two treatment groups using the randomization tool from the Research Electronic Data Capture (RedCap) system.

b. The solution will be warmed in the front upper pocket of an anesthesia provider for at least one hour prior to administration. The patient will not receive any medication that has not been warmed for at least one hour. The anesthesiologist who is warming the medication will always be immediately available and the medication will be distributed as soon as it is needed.

c. Room temperature medication – epidural bolus of 10 ml of 0.125% Bupivacaine with Fentanyl 2mcg/ml given in 10 mL doses given by an anesthesia provider not involved in patient assessment. 'Time 0' will be considered the time the last epidural dose is administered.

i. Temperature (°C) of 10mL syringe of normal saline that is stored with the room temperature epidural medication syringe will be measured with an STS-400 temperature sensor (Smiths Medical, Rockland, MA, USA). Note that directly measuring the temperature of the epidural medications to be administered to the patient is not sterile and thus not advisable, thus we intend to measure the temperature of a separate normal saline syringe of the same volume and stored in the same environment as the medication administered to the patient.

1. Note that we have measured the temperature of two separate 0.9% normal saline test syringes stored in the same room temperature location, and test syringes consistently measure the same (within the accuracy limits of the STS-400 temperature sensor). The bupivacaine + fentanyl epidural medications are diluted in sterile 0.9% normal saline, so the temperature of the 0.9% normal saline test syringes that are measured are expected to accurately reflect the temperature of the epidural medication that is administered to the patient.

d. Pocket warmed medication – 10 ml of 0.125% Bupivacaine with Fentanyl 2mcg/ml stored within a clean plastic bag in the front pocket of an anesthesia provider for a minimum of 1 hour prior to administration. The epidural medication will be given in 10 mL doses given by an anesthesia provider not involved in patient assessment. 'Time 0' will be considered the time the last epidural dose is administered.

i. Temperature (°C) of 10mL normal saline syringe that is stored in the warm pocket environment with epidural medication syringe will be measured with an STS-400 temperature sensor (Smiths Medical, Rockland, MA, USA).

1. Note that we have measured the temperature of 0.9% normal saline test syringes stored in the same pocket location, and they consistently measure very similarly. The bupivacaine + fentanyl epidural medications are diluted in sterile 0.9% normal saline, so the temperature of the 0.9% normal saline test syringes that are measured are expected to accurately reflect the temperature of the epidural medication that is administered to the patient.

e. If the patient does not obtain satisfactory analgesia after 15 minutes, then an additional 5mL of epidural medication will be administered (room temperature or pocket warmed medication,

according to study group; note that additional room temperature and pocket warmed medication will be immediately available if it is required)

f. If the patient still has inadequate analgesia after 5 additional minutes, then an additional 5mL of epidural medication will be administered (room temperature or pocket warmed medication, according to study group)

5) Maintenance of labor analgesia:

a. All patients will receive a continuous epidural infusion of 0.0625% Bupivacaine with 2mcg/ml Fentanyl at 10 mL given every 45-60 minutes with a patient-controlled epidural analgesia (PCEA) available of 8 mL every 10 minutes and a 40mL hourly lockout.

6) Outcomes, Variables, and Endpoints:

a. Primary outcome:

i. Time until a VRS $\leq$ 3 - VRS pain scores will be assessed and recorded immediately after completion of the initial 10mL epidural bolus (time 0), and at 5, 10, 15, 20, 30 and 60 minutes. The time will also be recorded at the end of the first uterine contraction with VRS $\leq$3.

1. A definition of VRS $\leq$ 3 was chosen as a definition for adequate labor analgesia to maintain consistency with the only previous study on administering warmed epidural medication to laboring patients.[1]

b. Secondary outcomes that will be recorded:

i. Patient satisfaction regarding initiation of labor analgesia will be assessed by a 1-100 rating scale on postpartum day #1 during the routine follow-up visit.

ii. Oral temperature will be assessed and recorded immediately after completion of the initial 10mL epidural bolus (time 0) and again at 15, 30, 60 minutes.

iii. Fever (oral temperature >38 °C) anytime during labor will be recorded

iv. Presence of nausea, vomiting, shivering, and blood pressure will be assessed and recorded immediately after completion of the initial 10mL epidural bolus (time 0) and again at 5, 10, 15, 20, 30 and 60 minutes.

v. Management of breakthrough pain during labor epidural analgesia will also be recorded, including:

1. Required rescue epidural bolus (yes/no)

2. Time to first manual epidural rescue bolus (minutes)

3. Total number of manual anesthetic interventions to treat inadequate labor analgesia

vi. Mode of delivery (cesarean, spontaneous vaginal or instrumental vaginal delivery)

c. Endpoints:

i. Epidural failure – if the patient never achieves adequate labor analgesia from a labor epidural placement.

ii. Vaginal Delivery – whether spontaneous or assisted vaginal delivery

iii. Cessation of labor analgesia due to obstetric decision to proceed with cesarean delivery

7) Subject Safety, Potential Benefit and Privacy

a. Risks: Initiation of labor analgesia with either room temperature local anesthetic medications or warmed medications are considered safe techniques based on the available evidence.

i. There may be concern for higher incidence of fever after administering warmed local anesthetic medication, though the only previous study done in laboring patients did not observe any difference among groups in incidence of fever or rise in patient temperature.[1] It should be noted here that these authors warmed the solution to 37 °C[1] and previously we have documented that pocket warming is only to approximately 29.7 °C.[17]

ii. There may also be concern for higher incidence of nausea, vomiting, and hypotension after administering warmed local anesthetic medication. Once again, the only previous study done in laboring patients did not observe any difference among groups in incidence of nausea, vomiting, and hypotension.[1]

iii. There may be concern for greater incidence of shivering may occur in patients receiving room temperature medication compared to pocket warmed medication, though statistically significant differences were not observed in the previous study. [1]

b. Potential Benefits: Initiation of labor analgesia with either room temperature local anesthetic medications or warmed medications are considered safe and effective techniques. Initiation of labor analgesia with pocket warmed epidural medications has a few potential advantages:

i. There is potential to achieve a more rapid onset of labor analgesia (adequate analgesia defined as VRS $\leq$ 3) when dosing with warmed epidural medications. [1]

ii. There is potential to improve patient satisfaction when dosing with pocket warmed epidural medications due to the more rapid onset of labor analgesia.

iii. There is potential to reduce incidence and severity of shivering when dosing with pocket warmed epidural medications relative to room temperature medications, though statistically significant differences were not observed in the previous study. [1]

c. Privacy:

i. All data collected in this study will be stored on computer systems that require user authentication for logon. After data collection is complete, none of this electronic data will be stored with subject-identifying information and will be archived and kept indefinitely. All written records or consent forms that identify the subject will be stored in a locked file.

D. Statistical Considerations

Sample Size:

A total of 62 patients in each group we would have 90% power to detect a 5 minute difference. We intend to include 75 patients in each group to allow for patients that may not obtain adequate labor analgesia at any time after epidural placement and administration of medication. Note that a previous study from an academic practice reported an estimate of 6.8% of patients may not obtain adequate labor analgesia after epidural placement.[4]

Primary Analysis:

To compare the overall effect of temperature on pain relief the number of minutes between epidural placement and VRS $\leq$3 will be compared between the two treatment groups. Regression models will be utilized including treatment and baseline pain scores as predictor variables; Cox-proportional hazards models will be used if censoring is observed. Sensitivity analyses may be performed to adjust for clinically relevant covariates and/or any differences observed in baseline summaries.

Secondary Analysis:

Descriptive statistics will be generated for each secondary outcome, both overall and by treatment arm. Regression models will be used to compare secondary outcomes between study arms.

Interim Analysis:

No formal interim analysis for futility or efficacy is planned. However, adverse events will be continuously monitored and the trial may be stopped if concern for patient safety arises.

Demographic and Baseline Characteristics:

Demographic and baseline characteristics will be summarized for all patients registered to the study. Results will be summarized by treatment arm. Continuous responses will be summarized using means, medians and other appropriate measures of spread. Categorical responses will be summarized using frequencies and percentages.

IV. Bibliography/References

1. Sviggum HP, Yacoubian S, Liu X, Tsen LC. The effect of bupivacaine with fentanyl temperature on initiation and maintenance of labor epidural analgesia: a randomized controlled study. *Int J Obstet Anesth* 2015; 24:15-21.
2. Attanasio L, Kozhimannil KB, Jou J, McPherson ME, Camann W. Women's Experiences with Neuraxial Labor Analgesia in the Listening to Mothers II Survey: A Content Analysis of Open-Ended Responses. *Anesth Analg* 2015; 121: 974-80.
3. Gambling D, Berkowitz J, Farrell TR, Pue A, Shay D. A randomized controlled comparison of epidural analgesia and combined spinal-epidural analgesia in a private practice setting: pain scores during first and second stages of labor and at delivery. *Anesth Analg* 2013 Mar;116(3):636-43.
4. Pan PH, Bogard TD, Owen MD. Incidence and characteristics of failures in obstetric neuraxial analgesia and anesthesia: a retrospective analysis of 19,259 deliveries. *Int J Obstet Anesth* 2004;13: 227-33.
5. Riley ET, Hamilton CL, Ratner EF, Cohen SE. A comparison of the 24-gauge Sprotte and Gertie Marx spinal needles for combined spinal-epidural analgesia during labor. *Anesthesiology* 2002; 97: 574-7.
6. Van de Velde M, Vercauteren M, Vandermeersch E. Fetal heart rate abnormalities after regional analgesia for labor pain: the effect of intrathecal opioids. *Reg Anesth Pain Med.* 2001; 26: 257-62.
7. Mardirosoff C, Dumont L, Boulvain M, Tramèr MR. Fetal bradycardia due to intrathecal opioids for labour analgesia: a systematic review. *BJOG* 2002; 109: 274-81.
8. Abrão KC, Francisco RP, Miyadahira S, Cicarelli DD, Zugaib M. Elevation of uterine basal tone and fetal heart rate abnormalities after labor analgesia: a randomized controlled trial. *Obstet Gynecol* 2009; 113: 41-7.
9. Cook TM, Counsell D, Wildsmith JA; Royal College of Anaesthetists Third National Audit Project. Major complications of central neuraxial block: report on the Third National Audit Project of the Royal College of Anaesthetists. *Br J Anaesth* 2009; 102: 179-90.
10. Gaiser RR, McHugh M, Cheek TG, Gutsche BB. Predicting prolonged fetal heart rate deceleration following intrathecal fentanyl/bupivacaine. *Int J Obstet Anesth* 2005; 14: 208-11.

11. Mehta PM, Theriot E, Mehrotra D, Patel K, Kimball BG. A simple technique to make bupivacaine a rapid acting epidural anesthetic. *Reg Anesth* 1987; 12:135-8.

12. Santagostino G, Messeri E, Pezzati S, Gambi D. Peridural anesthesia with 0.5% bupivacaine-fentanyl, Evaluation of the pre-heating effect. *Minerva Anestesiol* 1992; 58:433-6.

13. Liu F, Liou J, Day Y, Li A, Yu H. Effect of warm lidocaine on the sensory onset of epidural anesthesia: a randomized trial. *Chang Gung Med J* 2009; 32: 643-9.

14. Clark V, McGrady E, Sugden C, Dickson J, McLeod G. Speed of onset of sensory block for elective extradural cesarean section: choice of agent and temperature of injectate. *Br J Anaesth* 1994; 72:221-3.

15. Beardsworth D, Lambert DH. Warming 0.5% bupivacaine to 37 decrees C increases duration of spinal anesthesia. *Reg Anesth* 1989; 14:199-202.

16. Lee R, Kim YM, Choi EM, Choi YR, Chung MH. Effect of warmed ropivacaine solution on onset and duration of axillary block. *Korean J Anesthesiol* 2012; 62: 52–56.

17. Coffman JC, Fiorini K, Mathew P, Small RH. Pocket warming of epidural medications. *Int J Obstet Anesth* 2015; 24: 290-1.

18. Material Safety Data Sheet for Hospira Bupivacaine Hydrochloride. Available at: https://www.amatheon.com/msds/Bupivacaine_HCL_Hospira.pdf. Accessed January 21, 2016.
