# Supplementary material for: Pocket warming of bupivacaine with fentanyl to shorten onset of labor epidural analgesia: A double-blind randomized controlled clinical trial
Source: PLoS One. 2024 Sep 4;19(9):e0309515. doi: 10.1371/journal.pone.0309515 (PMC11373790; doi:10.1371/journal.pone.0309515)
Supplement: Attachment — Submitted filename: PONE-D-24-05704_R1_15 JUl.pdf [file pone.0309515.s006.pdf]

PLOS ONE

Pocket Warming of Bupivacaine with Fentanyl to Shorten Onset of Labor Epidural Analgesia: A Double-Blind Randomized Controlled Clinical Trial

--Manuscript Draft--

**Manuscript Number:**	PONE-D-24-05704R1
**Article Type:**	Clinical Trial
**Full Title:**	Pocket Warming of Bupivacaine with Fentanyl to Shorten Onset of Labor Epidural Analgesia: A Double-Blind Randomized Controlled Clinical Trial
**Short Title:**	Pocket Warming Medications to Shorten Onset of Labor Epidural Analgesia
**Corresponding Author:**	Ling-Qun Hu, MD<br>The Ohio State University College of Medicine<br>Columbus, OH UNITED STATES
**Keywords:**	Prewarming medication, temperature of medication, labor analgesia onset, bupivacaine onset time, initiation of epidural analgesia
**Abstract:**	Shortening analgesic onset has been researched and it has been documented that prewarming epidural medications to body temperature (37℃) prior to administration increases medication efficacy. Our double-blind randomized controlled trial was designed to investigate if a lower degree of prewarming in providers' pockets could achieve similar results without the need of a bedside incubator. A total of 136 parturients were randomized into either the pocket-warmed group or the room temperature group to receive 10 mL of 0.125% bupivacaine with 2 μg/mL fentanyl epidural bolus at either the 27.8 ±1.7℃ or 22.1 ±1.0℃ temperatures, respectively. Primary outcome, time to analgesic onset (verbal rating scale pain score ≤ 3) was recorded in 0, 5, 10, 15-, 20-, 30-, and 60-minutes intervals. It was observed that the pocket-warming group (n = 64) and room temperature group (n = 72) had a similar analgesic onset time (median 8 vs. 6.2 minutes; p = 0.322). The incidence of adverse events such as hypotension, fever (≥ 38℃), nausea, vomiting, and number of rescue epidural boluses, as well as patient satisfaction rates, were similar between the groups as well.<br>Further research is warranted to confirm these findings and explore the impact of different temperatures on analgesic onset time as well as the logistical issues associated with their clinical implementations.
**Order of Authors:**	Tyler M. Balon, Medical student
	Yun Xia, MD, PhD
	Johnny McKeown, Medical student
	Jack Wang, Medical student
	Justin J. Abbott, Medical student
	Marilly Palettas, MPH
	Alberto Uribe, MD, MS, MPHc
	Marco Echeverria Villalobos, MD
	John C. Coffman, MD
	Ling-Qun Hu, MD
**Response to Reviewers:**	Minor revisions:<br>1- Line 110: Include the full details for the sample size justification. The power calculation should include: (1) the estimated outcomes in each group; (2) the α (type I) error level; (3) the statistical power (or the β (type II) error level); (4) the target sample size, (5) the statistical testing method, and (6) for continuous outcomes, the standard deviation of the measurements.<br>Addressed in the manuscript. "A total of 62 patients in each group would have 90% power to detect a 5-minute difference, using a one-way analysis of variance with α=0.05. These estimates were based on an effect size of 0.42 (μ1=4, μ2=9, σ=6). We

Powered by Editorial Manager® and ProduXion Manager® from Aries Systems Corporation

	intend to include 75 patients in each group (total estimated sample size of 150) to allow for patients that may not obtain adequate labor analgesia at any time after epidural placement and administration of medication. The number of patients was determined based on the sample size calculation as well as a 6.8% incidence of inadequate LEA that was previously reported at an academic center."<br><br>2- Line 121: If block randomization was used, state the block size.<br>Addressed in the manuscript – block size of 6 was used.<br><br>3- Line 154: Indicate the underlying covariance structure used in the mixed effects models and the criteria for selecting it.<br>Addressed in the manuscript – compound symmetry was the covariance structure used in the mixed effects model. No particular pattern in the correlation among repeated measure observations was seen when evaluating the residual plots.<br><br>4- The standard statistical term for average is mean.<br>Addressed in the manuscript.<br><br>5- Table 1: Provide only one p-value for comparing race. The one from the overall chi-square or Fisher's exact test.<br>Each category of race was captured as a separate variable and the proportions and p-values presented are the "Yes" vs "No" comparisons for each race category.<br><br>6- Line 208: Replace the two instances of the term "stratified" because it has a specific statistical meaning in relation to the randomization process which has not been conducted in the study. Perhaps "dichotomized" is a more descriptive term.<br>Addressed in the manuscript.<br><br>7- Line 216: Provide the overall p-value for testing the interaction effect of group by time. If the interaction effect is significant, provide an interpretation of the results, but do not test main effects because the tests for main effects are uninteresting in light of significant interactions. If interaction effects are non-significant, drop the interaction effects from the model and test the main effects. Determining which results to present when testing interactions is often a multi-step process.<br>Addressed in the manuscript – The overall p-value for the interaction effect of group by time was 0.418 and it was dropped from the model. Time was the only significant main effect ($p<0.001$).<br><br>8- Line 223: For clarification, indicate that the secondary outcomes (rate of adverse events) did not differ significantly between the groups.<br>Addressed in the manuscript.
**Additional Information:**	
**Question**	**Response**
**Financial Disclosure**<br><br>Enter a financial disclosure statement that describes the sources of funding for the work included in this submission. Review the submission guidelines for detailed requirements. View published research articles from *PLOS ONE* for specific examples.<br><br>This statement is required for submission and **will appear in the published article** if the submission is accepted. Please make sure it is accurate.	Yes

**Funded studies**<br>Enter a statement with the following details:<br>• Initials of the authors who received each award<br>• Grant numbers awarded to each author<br>• The full name of each funder<br>• URL of each funder website<br>• Did the sponsors or funders play any role in the study design, data collection and analysis, decision to publish, or preparation of the manuscript?<br><br>Did you receive funding for this work?	
Please add funding details.<br>as follow-up to "**Financial Disclosure**<br><br>Enter a financial disclosure statement that describes the sources of funding for the work included in this submission. Review the submission guidelines for detailed requirements. View published research articles from *PLOS ONE* for specific examples.<br><br>This statement is required for submission and **will appear in the published article** if the submission is accepted. Please make sure it is accurate.<br><br>**Funded studies**<br>Enter a statement with the following details:<br>• Initials of the authors who received each award<br>• Grant numbers awarded to each author<br>• The full name of each funder<br>• URL of each funder website<br>• Did the sponsors or funders play any role in the study design, data collection and analysis, decision to publish, or preparation of the manuscript?<br><br>Did you receive funding for this work?"	1) Medical Student Research (MDSR) Roessler Scholarship from The Ohio State University – College of Medicine:<br>Winters:<br>Tyler M. Balon: data collection, drafting<br>Justin J. Abbott: data collection<br>URL: https://medicine.osu.edu/research/opportunities/mdsr-resources/apply-for-medical-student-research-scholarship<br><br>2) The Foundation of Anesthesia Education and Research (FAER)'s Medical Student Anesthesia Research Fellowships<br>Winner: Johnny McKeown, data collection, drafting;<br>URL:<br>https://www.asahq.org/faer/programs/medicalstudentfellowships/summerfellowships
Please select the country of your main research funder (please select carefully as in some cases this is used in fee calculation).	UNITED STATES - US

as follow-up to "**Financial Disclosure**<br><br>Enter a financial disclosure statement that describes the sources of funding for the work included in this submission. Review the submission guidelines for detailed requirements. View published research articles from *PLOS ONE* for specific examples.<br><br>This statement is required for submission and **will appear in the published article** if the submission is accepted. Please make sure it is accurate.<br><br>**Funded studies**<br>Enter a statement with the following details:<br>• Initials of the authors who received each award<br>• Grant numbers awarded to each author<br>• The full name of each funder<br>• URL of each funder website<br>• Did the sponsors or funders play any role in the study design, data collection and analysis, decision to publish, or preparation of the manuscript?<br><br>Did you receive funding for this work?"	
**Competing Interests**<br><br>Use the instructions below to enter a competing interest statement for this submission. On behalf of all authors, disclose any competing interests that could be perceived to bias this work—acknowledging all financial support and any other relevant financial or non-financial competing interests.<br><br>This statement is required for submission and **will appear in the published article** if the submission is accepted. Please make sure it is accurate and that any funding sources listed in your Funding Information later in the submission form are also declared in your Financial Disclosure statement.	The authors have declared that no competing interests exist.

View published research articles from *PLOS ONE* for specific examples.<br><br>**NO authors have competing interests**<br><br>Enter: *The authors have declared that no competing interests exist.*<br><br>**Authors with competing interests**<br><br>Enter competing interest details beginning with this statement:<br><br>*I have read the journal's policy and the authors of this manuscript have the following competing interests: [insert competing interests here]*<br><br>* typeset	
**Ethics Statement**<br><br>Enter an ethics statement for this submission. This statement is required if the study involved:<br><br>• Human participants<br>• Human specimens or tissue<br>• Vertebrate animals or cephalopods<br>• Vertebrate embryos or tissues<br>• Field research<br><br>Write "N/A" if the submission does not require an ethics statement.<br><br>General guidance is provided below. Consult the submission guidelines for detailed instructions. **Make sure that all information entered here is included in the Methods section of the manuscript.**	Office of Responsible Research Practices (ORRP)—The Ohio State University in 2016 (Protocol ID: 2016H0153).<br>Written Informed Consents were obtained in all the study subjects.

**Format for specific study types**<br><br>**Human Subject Research (involving human participants and/or tissue)**<br>• Give the name of the institutional review board or ethics committee that approved the study<br>• Include the approval number and/or a statement indicating approval of this research<br>• Indicate the form of consent obtained (written/oral) or the reason that consent was not obtained (e.g. the data were analyzed anonymously)<br><br>**Animal Research (involving vertebrate animals, embryos or tissues)**<br>• Provide the name of the Institutional Animal Care and Use Committee (IACUC) or other relevant ethics board that reviewed the study protocol, and indicate whether they approved this research or granted a formal waiver of ethical approval<br>• Include an approval number if one was obtained<br>• If the study involved *non-human primates*, add *additional details* about animal welfare and steps taken to ameliorate suffering<br>• If anesthesia, euthanasia, or any kind of animal sacrifice is part of the study, include briefly which substances and/or methods were applied<br><br>**Field Research**<br><br>Include the following details if this study involves the collection of plant, animal, or other materials from a natural setting:<br>• Field permit number<br>• Name of the institution or relevant body that granted permission	
**Data Availability**<br><br>Authors are required to make all data underlying the findings described fully available, without restriction, and from the time of publication. PLOS allows rare exceptions to address legal and ethical concerns. See the PLOS Data Policy and FAQ for detailed information.	Yes - all data are fully available without restriction

A Data Availability Statement describing where the data can be found is required at submission. Your answers to this question constitute the Data Availability Statement and **will be published in the article**, if accepted.<br><br>**Important:** Stating 'data available on request from the author' is not sufficient. If your data are only available upon request, select 'No' for the first question and explain your exceptional situation in the text box.<br><br>Do the authors confirm that all data underlying the findings described in their manuscript are fully available without restriction?	
**Describe where the data may be found in full sentences. If you are copying our sample text, replace any instances of XXX with the appropriate details.**<br><br>• If the data are **held or will be held in a public repository**, include URLs, accession numbers or DOIs. If this information will only be available after acceptance, indicate this by ticking the box below. For example: *All XXX files are available from the XXX database (accession number(s) XXX, XXX.*).<br>• If the data are all contained **within the manuscript and/or Supporting Information files**, enter the following: *All relevant data are within the manuscript and its Supporting Information files.*<br>• If neither of these applies but you are able to provide **details of access elsewhere**, with or without limitations, please do so. For example:<br><br>*Data cannot be shared publicly because of [XXX]. Data are available from the XXX Institutional Data Access / Ethics Committee (contact via XXX) for researchers who meet the criteria for access to confidential data.*<br><br>*The data underlying the results presented in the study are available from (include the name of the third party*	All relevant data are within the manuscript and its Supporting Information files.

and contact information or URL).<br>• This text is appropriate if the data are owned by a third party and authors do not have permission to share the data.<br><br>* typeset	
Additional data availability information:	Tick here if the URLs/accession numbers/DOIs will be available only after acceptance of the manuscript for publication so that we can ensure their inclusion before publication.

Manuscript without tracking

Click here to access/download;Manuscript;Pocket Warming PaperFINAL_11FEB2024 MP(unmarked version).docx

Full Title:
Pocket Warming of Bupivacaine with Fentanyl to Shorten Onset of Labor Epidural Analgesia: A Double-Blind Randomized Controlled Clinical Trial

Shortened Title:
Pocket Warming Medications to Shorten Onset of Labor Epidural Analgesia

Authors: Tyler M. Balon[1], Yun Xia[2], Johnny McKeown[3], Jack Wang[1], Justin J. Abbott[1], Marilly Palettas[4], Alberto Uribe[2], Marco Echeverria Villalobos[2], John C. Coffman[2], Ling-Qun Hu[2]

Affiliations:
1. The Ohio State University, College of Medicine, Columbus, Ohio
2. The Ohio State University Wexner Medical Center, Department of Anesthesiology, Columbus, Ohio
3. The University of Toledo, College of Medicine and Life Sciences, Toledo, Ohio
4. The Ohio State University, College of Medicine, Center for Biostatistics, Columbus, Ohio

Corresponding Author:

Ling-Qun Hu, MD
The Ohio State University Wexner Medical Center-Department of Anesthesiology
410 W. 10th Ave. N411
Columbus, Ohio 43210
Office: (614) 293-3559
Fax: (614) 366-1943
Email: LingQun.Hu@osumc.edu

These authors also contributed equally to this work

Abbreviations:
LEA: Labor epidural analgesia
VRS: Verbal Rating Scale
IQR: interquartile ranges
N: number of participants
%: percentage
CI: Confidence interval
SD: standard deviation
PIEB: programmed intermittent epidural bolus
MDSR: Medical Student Research
FAER: Foundation for Anesthesia Education and Research

Abstract:

Shortening analgesic onset has been researched and it has been documented that prewarming epidural medications to body temperature (37℃) prior to administration increases medication efficacy. Our double-blind randomized controlled trial was designed to investigate if a lower degree of prewarming in providers' pockets could achieve similar results without the need of a bedside incubator. A total of 136 parturients were randomized into either the pocket-warmed group or the room temperature group to receive 10 mL of 0.125% bupivacaine with 2 µg/mL fentanyl epidural bolus at either the 27.8 ±1.7℃ or 22.1 ±1.0℃ temperatures, respectively. Primary outcome, time to analgesic onset (verbal rating scale pain score ≤ 3) was recorded in 0-, 5-, 10-, 15-, 20-, 30-, and 60-minutes intervals. It was observed that the pocket-warming group (n = 64) and room temperature group (n = 72) had a similar analgesic onset time (median 8 vs. 6.2 minutes; p = 0.322). The incidence of adverse events such as hypotension, fever (≥ 38℃), nausea, vomiting, and number of rescue epidural boluses, as well as patient satisfaction rates, were similar between the groups as well.

Further research is warranted to confirm these findings and explore the impact of different temperatures on analgesic onset time as well as the logistical issues associated with their clinical implementations.

Introduction:

Labor epidural analgesia (LEA) is a widely accepted practice and is a safe and effective method to control pain during labor, usually taking only 10-20 minutes for analgesic onset to occur [1]. To achieve more rapid onset of LEA, researchers and physicians have tried strategies such as utilizing faster-acting local anesthetics such as chloropropane, employing higher concentration agents, adding various adjuvants such as sodium bicarbonate, or using other neuraxial approaches such as combined-spinal epidural and dural puncture epidural [2]. Warming the initial dose of epidural medications to 37℃ prior to administration has been studied as well, and it was shown to shorten the onset of action of the medication by more than 5 minutes without lowering its analgesic properties [3]. However, the use of expensive incubators to warm controlled substances in labor and delivery rooms may not be practical in every clinical setting.

Could providers replicate this effect by prewarming the initial epidural doses in their pockets, albeit to a lesser temperature, to achieve a similar result? [4] If so, it would be expected to be less expensive and readily available to every obstetric anesthesia service.

Our null hypothesis is that there is no difference in onset of LEA between patients who receive either a pocket-warmed or room temperature bolus of bupivacaine with fentanyl.

Materials and methods:

This is a prospective, double-blind, randomized clinical trial. The study protocol was approved by approval from our local IRB, Office of Responsible Research Practices (ORRP)—The Ohio State University in 2016 (Protocol ID: 2016H0153) and registered at ClinicalTrails.gov (NCT02912078). The principal investigator was changed on January 26, 2022. Written Informed Consents were obtained in all the study subjects. The primary outcome is onset time of Verbal Rating Scale (VRS) pain score ≤ 3 after the initial LEA dose. A total of 62 patients in each group would have 90% power to detect a 5-minute difference, using a one-way analysis of variance with α=0.05. These estimates were based on

an effect size of 0.42 (μ_1=4, μ_2=9, σ=6). We intend to include 75 patients in each group (total estimated sample size of 150) to allow for patients that may not obtain adequate labor analgesia at any time after epidural placement and administration of medication. The number of patients was determined based on the sample size calculation as well as a 6.8% incidence of inadequate LEA that was previously reported at an academic center [5]. Inclusion criteria included women requesting LEA with a single vertex presentation fetus at term, with either intact fetal membranes or membrane rupture <6 hours previously. Exclusion criteria included patients with chronic pain, allergies or significant adverse reactions to routinely used local anesthetics or opioids, a contraindication to epidural placement, a baseline temperature over 38°C, or any patient that did not speak English, was incarcerated, or less than 18 years of age.

Our research team was divided into unblinded and blinded personnel. After screening and enrolling patients, unblinded investigators used REDCap to randomize subjects. Block randomization with a block size of 6 was used as the randomization scheme. Both the study subjects and data collecting researchers were blinded from the study groups. A designated researcher collected temperature data alone, was blinded from all other data, and was not present in patients' rooms. An unblinded anesthesiologist, was responsible for randomizing patients, administering medications, measuring temperatures, and subsequently reporting temperature data to the designated unblinded researcher without disclosing any other data. Finally, additional blinded team members were responsible for collecting all other data such as the primary and secondary outcomes.

Syringes containing 10 mL epidural 0.125% bupivacaine with fentanyl 2 µg/mL were pre-made and stored in the anesthesia workroom of the labor and delivery unit. The study medications were checked out ahead of time by investigating anesthesiologists and kept in the upper pockets of their surgical scrubs for at least 1 hour together with a 10 mL commercially available saline syringe. For the pocket warmed group, the medication temperature was obtained by measuring the accompanying saline syringe. For the room temperature group, medication temperature was recorded from a saline syringe stored on a shelf at the same altitude level in the same room. For both groups, disposable skin

temperature sensors (DeRoyal Industries Inc, 200 DeBusk Lane, Powell, TN USA) with GE anesthesia monitors were used to measure temperature immediately following administration of epidural medications.

After screening, parturients were randomized to receive epidural medications that had either been pocket warmed or stored at room temperature. The onset of adequate labor analgesia, defined as pain VRS $\leq$ 3, was assessed at 0-, 5-, 10-, 15-, 20-, 30-, and 60-minutes post initial epidural dose. Secondary outcomes included shivering, nausea, vomiting, temperature > 38 ℃, hypotension, number of rescue epidural boluses, and overall patient satisfaction assessed within 24 hours of delivery.

STATISTICAL ANALYSIS:

Patient demographic and clinical characteristics were summarized for the two study groups using appropriate descriptive statistics. Means with standard deviations or medians with interquartile ranges (IQR) were used for continuous variables, and frequencies and proportions were used for categorical variables. Comparisons between the room temperature and pocket warming groups included maternal demographics, gestational age, pregnancy history, mode of delivery, analgesic onset, VRS pain scores, and other outcomes. Categorical variables were compared between groups using either a Chi-square test or a Fisher's Exact test, and continuous variables were compared using either a two-sample t-test or a Wilcoxon Rank Sum test. The primary outcome of analgesic onset time was presented as median and IQR and compared between groups using Wilcoxon Rank Sum test. The incidence of VRS score ≤ 3/10 within 15 minutes was summarized using frequencies and proportions and compared between groups using a Chi-square test. Mixed-effects linear regression was used to evaluate differences in VRS pain scores between room temperature and pocket warming groups for the initial 60 minutes after epidural placement. Group and time were included as fixed effects in the model, and patients were included as a random effect to account for the correlation between repeated measures on the same patient. Compound symmetry was used as the covariance structure in the model given there was no correlation among repeated measure observations when evaluating the residual plots. Secondary outcomes (number of rescue epidural bolus, shivering, nausea and/or

vomiting, hypotension, fever, and patient satisfaction) were compared between the two groups using either a Chi-square test, Fisher's Exact test, two-sample t-test, or a Wilcoxon Rank Sum test, where appropriate. A value of P less than 0.05 was used to indicate statistical significance. Statistical analyses and plots were performed using SAS 9.4 (SAS Institute, Inc.; Cary, North Carolina, USA).

Results:

The study was conducted between November 2016 to August 2023. A total of 149 patients were consented and subsequently enrolled in the study, however 13 patients were excluded due to not meeting full inclusion criteria (n = 1), meeting one or more exclusion criteria (n = 2), having a baseline VRS < 3 (n = 4), having a cervical dilation over 8cm (n = 1), randomization error (n = 2), or due to a procedural deviation such as having a failed epidural that required replacement (n = 3). Therefore, a total of 136 women were analyzed. Of all eligible participants, 64 women were randomized to the pocket warming group, and 72 women were randomized to the room temperature group. The Consolidated Standards of Reporting Trials (CONSORT) flow diagram is shown in Fig 1 [6].

Fig 1. CONSORT Flow Diagram for Patients Enrolled in the study.

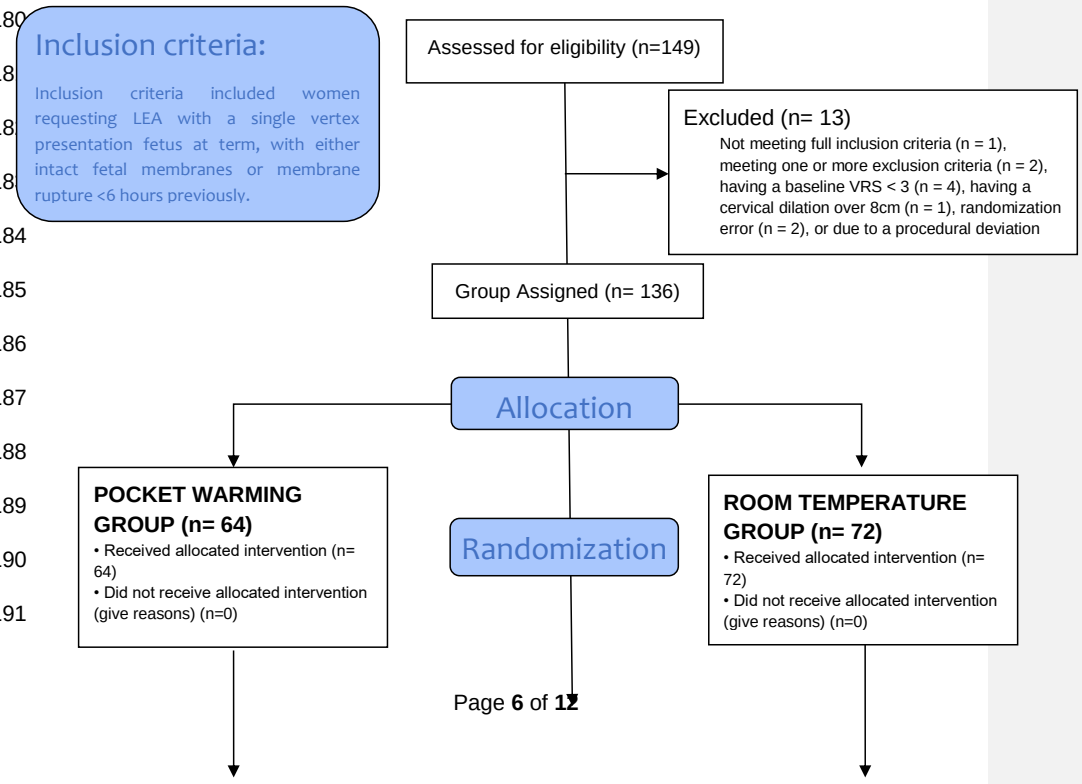

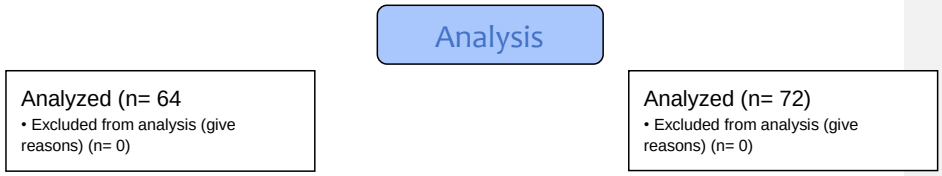

The two groups were demographically similar with no observed differences between race, age, BMI, gestational age, previous history of epidurals for laboring pain, cervical dilation at initiation of labor analgesia, and initial VRS pain score prior to epidural administration **(Table 1).** It was determined that the pocket warmed epidural (27.8±1.7℃) was on average 5.7 °C higher than the room temperature epidural (22.1±1.0℃). The study medications were observed to reach their maximal temperature following one hour of pocket warming, with any additional duration of warming not being associated with significant increases in medication temperature (P=0.405).

Table 1. Demographic Summary by Group Assignment

Variable	Room Temp. (n=72)	Pocket Warming (n=64)	P Values
Age (mean (SD))	31.6 (5.3)	30 (4.8)	0.067
Race			
White or Caucasian (n (%))	55 (76)	47 (73)	0.697
Black or African American (n (%))	13 (18)	15 (23)	0.525
Asian (n (%))	3 (4)	0 (0)	0.247
Body Mass Index (kg/m^2, mean (SD))	32.6 (7.1)	33.1 (6.5)	0.665
Gestational Age (weeks, mean (SD))	39.5 (0.7)	39.5 (0.8)	0.971
History of Labor Epidural in Prior Pregnancy, (n (%))			0.944
Multiparas	9 (13)	9 (14)	
Nulliparas	30 (42)	25 (39)	
Gravidity (median [IQR])	2 [1, 4]	2 [1, 2]	0.023
Cervical Dilation at Initiation of Labor Analgesia (cm, median [IQR])	4 [3, 4]	4 [2.5, 5]	0.668
Initial Pain Score (VRS, median [IQR])	7 [5.8, 8]	7 [6, 8]	0.432
Mode of delivery (n (%))			0.692
Cesarean delivery	16 (22)	10 (16)	
Instrumental delivery	1 (1)	1 (2)	
Spontaneous vaginal delivery	55 (76)	53 (83)	

Formatted Table

Legends n, numbers; SD, standard deviation; IQR, interquartile range; VRS, Verbal Rating Score

Shown on Table 2, the two groups were observed to have a similar time to analgesic onset when dichotomized by their median time required to reach a VRS score ≤ 3/10 or when dichotomized by a VRS score ≤ 3/10 in the first 15 minutes.

Table 2. The Primary Outcomes Between Room Temperature and Pocket Warming Groups.

Variable	Room Temp. (n=72)	Pocket Warming (n=64)	P Values
Analgesic onset (minutes)			
Median [IQR]	6.2 [4, 15]	8 [4.1, 16]	0.322
Mean (SD)*	10.3 (11.5)	12.3 (12.4)	
VRS score ≤ 3/10 within 15 minutes (n (%))	59 (82)	50 (78)	0.457

*the data was non-normal distribution by Shapiro-Wilk test
Legends: n, numbers; IQR, interquartile range; SD, standard deviation; VRS, Verbal Rating Score

In Mixed Effect Regression Analysis, no difference was detected in pain scores between pocket warming and room temperature groups at any time points after initial bolus: (overall p-value group*time = 0.418) [mean difference (95% CI), p-value - 0 min: 0.2 (-0.5, 1.0), P = 0.547; 5 min: 0.6 (-0.1, 1.4), P = 0.112; 10 min: 0.2 (-0.6, 1.0), P = 0.338] **(Fig 2).**

Fig 2: Verbal Rating Scale (VRS) Score for Pain over Time in Minutes for the Initial 60 Minutes after Epidural Placement

Fig 2 Legend: VRS: Verbal Rating Score

The secondary outcomes (incomplete LEA, shivering, nausea/vomiting, hypotension, fever, and patient satisfaction) did not differ significantly between the groups.

Table 3. The secondary Outcomes between Room Temperature and Pocket Warming Groups.

Variable, n (%)	Room Temp. (n=72)	Pocket Warming (n=64)	P Values
Rescue epidural bolus (n (%))	31 (43)	23 (36)	0.397
Shivering (n (%))	26 (36)	20 (31)	0.55
Nausea and/or Vomiting (n (%))	12 (17)	12 (19)	0.75
Hypotension (SBP ≤ 100, n (%))	5 (7)	4 (6)	0.871
Fever (Temp >38 ℃, (n (%))	3 (4)	1 (2)	0.623
Patient Satisfaction (%, median [IQR])	100 [100, 100]	100 [100, 100]	0.903

Legend: n, numbers; SBP, systolic blood pressure; Temp, temperature; IQR, interquartile range

Discussion

A similar analgesic onset time from the initial epidural dose was observed in both the pocket warmed (27.8℃) and room temperature group (22.1℃). The results were analyzed with comparisons of median onset time, incidence of VRS score ≤ 3 within 15 minutes, and VRS for pain over time in minutes for the initial 60 minutes after epidural in Mix Effectiveness Analysis. Additionally, secondary outcomes were not statistically significant either.

These findings differed from the findings in a previous study conducted by Sviggum and et al. [3]. The observed difference in the intervention temperature, 37℃ in the previous study vs. 27.8℃ in this study may play an important role. This is most likely because the temperature of the medication was not warm enough to elicit any significant difference in outcomes. An acceptable temperature, 37℃, may only be attainable through the use of an incubator rather than through the use of providers' pockets, which only warmed medication to 27.8℃ on average. Notably, pocket warming medications longer than 1 hour did not result in a higher temperature. This raises the question of what temperature level will result in a significant difference in onset of LEA, and whether the method used to achieve such a temperature could be implemented in a feasible and widely accessible manner.

Likewise, another potential explanation for the difference in results between our findings and those of Sviggum's may be due to the initial dose size. While the same medication was used in both studies, Sviggum used 20mL (as compared to 10mL in this study) which is capable of carrying more heat and

would be expected to have a quicker onset time either due to higher drug mass or its heating capacity if the temperature were to play a role [7]. Despite using the same definition for onset time as Sviggum et al., our median analgesic onset time was 6.2 [4, 15] minutes for the room temperature group, which was quicker than Sviggum's' room temperature onset time of 16.0 ± 10.5 min [3]. In addition, the analgesic onset time in both of our study groups was shorter than 9.2 ± 4.7 min, which was the mean onset time in their prewarmed (37°C) group. It is noted that the analysis methods are different from each other. Time data is usually skewed, especially in small studies. We used and reported median and interquartile description with Wilcoxon Rank Sum test accordingly for comparisons after confirmation of its non-normal distribution with a Shapiro-Wilk test. Interestingly, our mean time (and SD) in minutes for the room temperature and pocket warming groups were 10.3 (11.5) vs 12.3 (12.4), respectively. As such, our routinely used room temperature medications would only take one minute longer of onset than the 37°C solutions if so [3]. It must also be acknowledged that, despite using the same time intervals as were used in previous studies to collect VRS scores, the true LEA onset time is difficult to obtain. Uterine contractions are not continuous and analgesic onset occurs gradually over time rather than at an easily measurable point. Our reporting of the incidence of VRS ≤ 3/10 in the first 15 minutes, the cut off based on Sviggum's study, is to attempt to minimize this uncertainty.

Nevertheless, our study did confirm that warming epidural medication to a mean temperature of 27.8°C prior to administration did not pose any harm to parturients with respect to our measured secondary outcomes, i.e. their body temperature, incidences of hypotension, shivering, nausea, vomiting, and inadequate LEA. This is similar to what has been shown in the previous study [3].

There are several limitations in our study, especially when considering that it took more than 7 years from protocol approval to ultimately finish enrolling patients. During the study period, clinical practice changed over time, one example being that our unit introduced the programmed intermittent epidural bolus (PIEB) pumps in late 2019. Several outcome improvements have been documented after the same PIEB pumps were employed at other institutes [8]. However, the PIEB pump is designed to improve the maintenance of LEA, rather than alter the initiation of LEA which was the primary focus

of our study. The primary difference between the two pumps is that the first dose of 10 mL 0.0625% bupivacaine with 2 μg/mL fentanyl starts to be delivered 45 minutes after our initial (study) dose over 5 minutes via the PIEB pumps whereas the same amount of the same medications started to be delivered at the rate of 10 mL/h over 60 minutes evenly via the previous continuous infusion pumps. Patients were not allowed to give themselves patient-controlled epidural analgesia bolus in the study period, i.e. the first 60 minutes. Therefore, this practice change did not change the total amount of medications administered in the study period. Presumably, if it did alter study results, it would occur in both groups evenly without impacts on overall findings. It is also true that our principal investigator changed over time as well as our research team members. The impact of these changes seem very minimal given the fact that the middle trial analysis, mainly based on the data with the old pumps and the previous research team, supported our presumptions [9]. Secondly, we assumed the room temperature was consistently 20℃ but it was 22.1 ± 1.0℃ by measurement, which resulted in us underestimating the temperature difference between the two groups. The study sample size calculation was impacted consequently. Therefore, we could not rule out the possibility of type II error for our study.

Conclusions

In conclusion, the pocket warming (27.8℃) of bupivacaine with fentanyl did not result in a shorter onset of analgesia when compared with that of room temperature (22.1℃) bupivacaine with fentanyl. Further study is needed to confirm the previous findings with more commonly used initial dose of bupivacaine with fentanyl or/and practical logistics, especially medication stabilities and their optimal storage period before clinical utilization.

Acknowledgements:

The authors gratefully acknowledge Mahmoud Abdel-Rasoul, MS, MPH; Meghan Cook, MD; Harriet Washington, MD; Robert Small, MD; Goran Ristev, MD; Blair H. Hayes, MD; Jason Hoang, BA, McKenna Carr, BS and Jeremy Reeves, BA for their support, writing and editing collaboration that greatly improved our research project and manuscript (without them, it would not be possible to accomplish this project).

References:

1. Jones L, Othman M, Dowswell T, Alfirevic Z, Gates S, Newburn M, et al. Pain management for women in labour: an overview of systematic reviews. Cochrane database of systematic reviews. 2012;(3). doi.org/10.1002/14651858.CD009234.pub2
2. Chau A, Tsen L. Neuraxial labor analgesia: Initiation techniques. Best Practice & Research Clinical Anaesthesiology. 2022;36:3-15.
3. Sviggum H, Yacoubian S, Liu X, Tsen L. The effect of bupivacaine with fentanyl temperature on initiation and maintenance of labor epidural analgesia: a randomized controlled study. International Journal of Obstetric Anesthesia. 2015;24:15-21.
4. Coffman JC, Fiorini K, Mathew P, Small RH. Pocket warming of epidural medications. International Journal of Obstetric Anesthesia. 2015;24:290-291.
5. Pan PH, Bogard T, Owen M. Incidence and characteristics of failures in obstetric neuraxial analgesia and anesthesia: a retrospective analysis of 19,259 deliveries. International journal of obstetric anesthesia. 2004;13:227-233.
6. Begg C, Cho M, Eastwood S, Horton R, Moher D, Olkin I, et al. Improving the quality of reporting of randomized controlled trials. The CONSORT statement. JAMA. 1996;276:637-639. .
7. Chan L, Lee B, Kee WN. A randomised double-blinded controlled trial of the effect of diluent volume on the efficacy of a single dose of epidural ropivacaine for labour analgesia. International Journal of Obstetric Anesthesia. 2006;15:201-205.
8. Carvalho B, George RB, Cobb B, McKenzie C, Riley ET. Implementation of programmed intermittent epidural bolus for the maintenance of labor analgesia. Anesthesia & Analgesia. 2016;123:965-971.
9. Wang J LH, Bush MJ, Periel L, Hoang J, Coffman J, Palettas M, Hayes BH, Xia Y, Uribe A, Hu LQ. . Pocket Warming of Medications to shorten onset of labor epidural analgesia: A double-blind randomized clinical trial. Ohio Society of Anesthesiologists 83 rd Annual Meeting, Columbus Ohio 2022.

Click here to access/download;Figure;Figure 1. CONSORT enrolling flow diagram.doc

Figure 1. CONSORT flow diagram for patients enrolled in the study

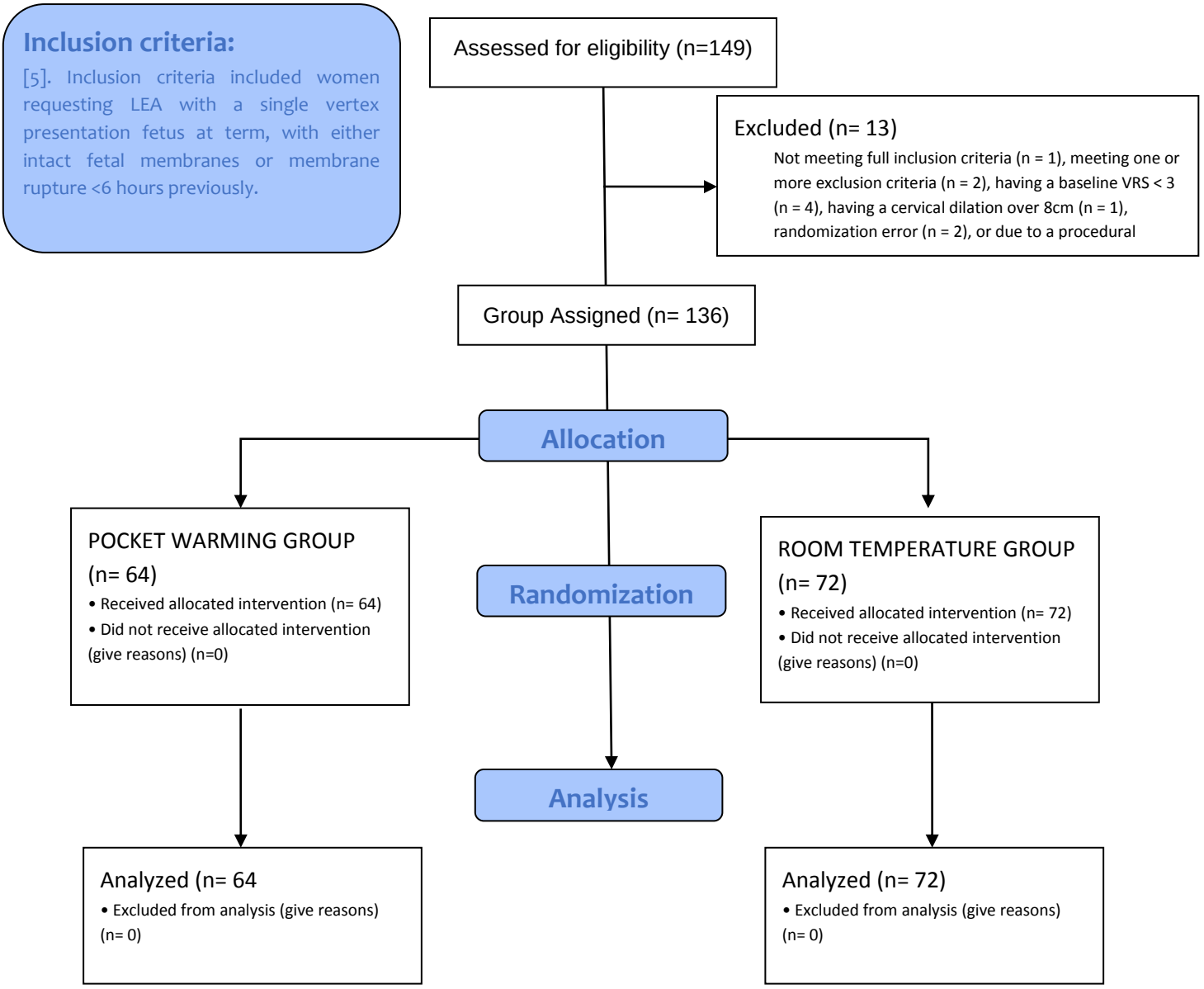

Legend: N; number of participants; MMA: multimodal analgesia

Figure 2

Click here to access/download;Figure;Figure 2..tiff

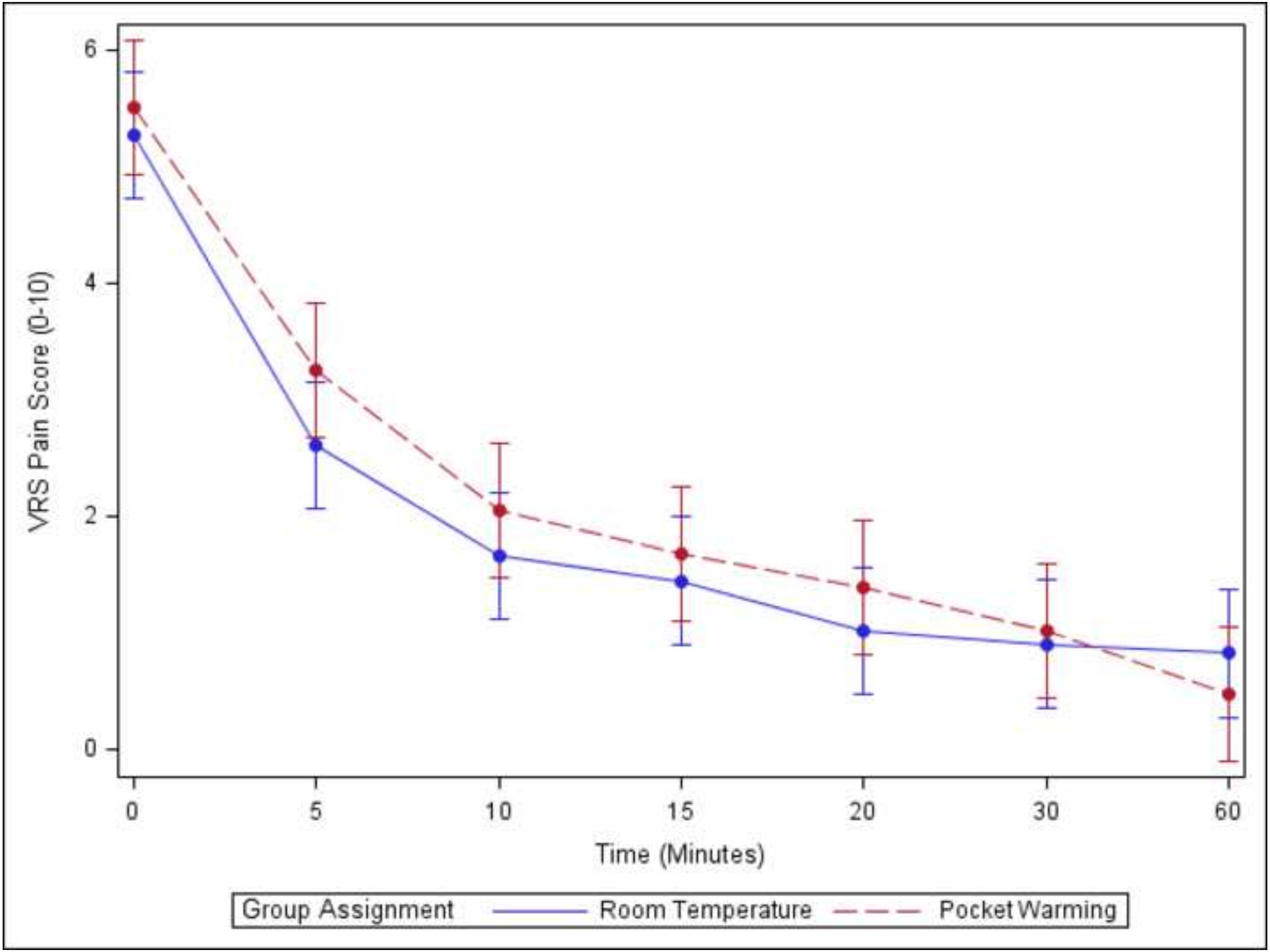

the CONSORT checklist

Click here to access/download
Supporting Information
CONSORT-2010-Checklist_13FEB2024.doc

the PDF copy of the protocol

Click here to access/download
Supporting Information
03_03_Pocket Warming_Research Protocol_22JUL2022.pdf

The ethics approval extension documents for the study.

Click here to access/download
Supporting Information
Initial & Continuing Approved for #2016H0153.pdf

Full Title:
Pocket Warming of Bupivacaine with Fentanyl to Shorten Onset of Labor Epidural Analgesia: A Double-Blind Randomized Controlled Clinical Trial

Shortened Title:
Pocket Warming Medications to Shorten Onset of Labor Epidural Analgesia

Authors: Tyler M. Balon[1], Yun Xia[2], Johnny McKeown[3], Jack Wang[1], Justin J. Abbott[1], Marilly Palettas[4], Alberto Uribe[2], Marco Echeverria Villalobos[2], John C. Coffman[2], Ling-Qun Hu[2]

Affiliations:
1. The Ohio State University, College of Medicine, Columbus, Ohio
2. The Ohio State University Wexner Medical Center, Department of Anesthesiology, Columbus, Ohio
3. The University of Toledo, College of Medicine and Life Sciences, Toledo, Ohio
4. The Ohio State University, College of Medicine, Center for Biostatistics, Columbus, Ohio

Corresponding Author:

Ling-Qun Hu, MD
The Ohio State University Wexner Medical Center-Department of Anesthesiology
410 W. 10th Ave. N411
Columbus, Ohio 43210
Office: (614) 293-3559
Fax: (614) 366-1943
Email: LingQun.Hu@osumc.edu

These authors also contributed equally to this work

Abbreviations:
LEA: Labor epidural analgesia
VRS: Verbal Rating Scale
IQR: interquartile ranges
N: number of participants
%: percentage
CI: Confidence interval
SD: standard deviation
PIEB: programmed intermittent epidural bolus
MDSR: Medical Student Research
FAER: Foundation for Anesthesia Education and Research
