# Supplementary material for: Pocket warming of bupivacaine with fentanyl to shorten onset of labor epidural analgesia: A double-blind randomized controlled clinical trial
Source: PLoS One. 2024 Sep 4;19(9):e0309515. doi: 10.1371/journal.pone.0309515 (PMC11373790; doi:10.1371/journal.pone.0309515)
Supplement: Attachment — Submitted filename: Rebuttal letter for Reviewer Comments_31July2024.docx.pdf [file pone.0309515.s007.pdf]

Dear Dr. Maged,

I am writing to address your comments and those of the reviewers, particularly Reviewer 4 and Reviewer 6, as detailed below. All the line information is based on the marked version.

Additionally, I have reorganized the parameter "mode of delivery" from Table 1 to Table 3 and have updated the corresponding text for better presentation.

For clarity, our responses are prefixed with "RESPONSE" and highlighted in purple.

Please let us know if you have any further questions or comments.

Sincerely,

Ling-Qun Hu, MD

Journal Requirements:

Please review your reference list to ensure that it is complete and correct. If you have cited papers that have been retracted, please include the rationale for doing so in the manuscript text, or remove these references and replace them with relevant current references. Any changes to the reference list should be mentioned in the rebuttal letter that accompanies your revised manuscript. If you need to cite a retracted article, indicate the article's retracted status in the References list and also include a citation and full reference for the retraction notice.

Reviewers' comments:

Reviewer's Responses to Questions

Comments to the Author

1. If the authors have adequately addressed your comments raised in a previous round of review and you feel that this manuscript is now acceptable for publication, you may indicate that here to bypass the "Comments to the Author" section, enter your conflict of interest statement in the "Confidential to Editor" section, and submit your "Accept" recommendation.

Reviewer #1: All comments have been addressed

Reviewer #3: All comments have been addressed

Reviewer #4: All comments have been addressed

Reviewer #5: All comments have been addressed

Reviewer #6: All comments have been addressed

2. Is the manuscript technically sound, and do the data support the conclusions?

Reviewer #1: (No Response)

Reviewer #3: Yes

Reviewer #4: Partly

Reviewer #5: (No Response)

Reviewer #6: Partly

3. Has the statistical analysis been performed appropriately and rigorously?

Reviewer #1: (No Response)

Reviewer #3: Yes

Reviewer #4: N/A

Reviewer #5: (No Response)

Reviewer #6: I Don't Know

4. Have the authors made all data underlying the findings in their manuscript fully available?

Reviewer #1: (No Response)

Reviewer #3: Yes

Reviewer #4: Yes

Reviewer #5: (No Response)

Reviewer #6: No

5. Is the manuscript presented in an intelligible fashion and written in standard English?

Reviewer #1: (No Response)

Reviewer #3: Yes

Reviewer #4: Yes

Reviewer #5: (No Response)

Reviewer #6: Yes

6. Review Comments to the Author

Reviewer #1: (No Response)

Reviewer #3: To authors,

First of all, I want to thank authors for this interesting review and for their efforts.

Title: Well written.

Abstract: well written

Background: is well written.

Methods: Thanks to authors as methods is well written in details.

Results:

• The data is statistically analyzed and expressed well.

• Tables and figures are expressed the data well.

Discussion: well written and relevant to the results of the study.

Conclusion: expressed the finding well and well written.

Best regard

Reviewer

Reviewer #4: i acknowledge the authors efforts in conducting this long duration trial. the subject is interested and of clinical importance which was conducted to explore if pocket warming would achieve similar results as body temperature warmed medications as compared to room temperature medications, however i have the following comments:

1- first it is better to describe your results (analgesia onset) by absence of significant difference between both groups and not describing them as similar results because there

was an observed difference in favor of room temperature group. - please correct it in abstract, results and discussion.

RESPONSE: Thank you for your comment. We have revised the manuscript based on your feedback as follows:

1. Line 63: "no significant difference of" has been used to replace "a similar."
2. Line 190: “not statistical differences” replaces “no observed differences”.
3. Line 201: “insignificant difference of” replaces “a similar time to”
4. Line 220: "A similar" has been replaced by "Absence of significantly different."

2- regarding the study outcomes, they needs more clarification regarding their definition- time points- end time point- method of assessment. for example the onset time was mentioned to be assessed for 60 minutes which doesn't reflect the clinical practice. onset of block is usually assessed every 5min within 30 min after the block if no adequate response extra-dose will be added. the time-point (60min) for follow up of the VRS score not the onset only.

RESPONSE: Thank you for requesting clarification. On Line 147-149, it was written: “The onset of adequate labor analgesia, defined as pain VRS < 3, was assessed at 0-, 5-, 10-, 15-, 20-, 30-, and 60-minutes post initial epidural dose,” reflecting the comment that “onset of block is usually assessed every 5 minutes within 30 minutes after the block if no adequate response, an extra dose will be added.” It is correct that the 60-minute time point is for follow-up of the VRS score, not the onset only, clinically. However, the 60-minute time point for following up the VRS score was our final timeline assessment to determine whether we needed to administer a rescue dose or replace the epidural catheter for this project, as detailed in the attached protocol. To clarify, “(the final VRS assessment)” is added immediately after “60-minutes” on Line 148, and “, the end point of the study” is added at the end of the sentence on Line 152.

and what did authors do if the analgesia target was not achieved .. they didn't exclude such cases.

when and how the rescue epidural doses were given? and why this outcome not reported in the results!

RESPONSE: Thank you for catching this and requesting clarification. Based on the protocol, we did have rescue doses or epidural replacements available to address unsuccessful epidural placements. We did have 3 incidences of replacements, and we updated the CONSORT chart accordingly. We also did not administer any rescue epidural doses. The “rescue doses” mentioned were actually top-off epidural doses that we routinely administer clinically to meet individual needs. These doses were not given until

60 minutes after the initial dose if the VRS was still elevated. Therefore, there was no need to exclude such cases from the analysis, but we did exclude 3 cases because of failed procedures. Accordingly, we have changed it as "Top-off epidural bolus" on Lines 150, 170, and 218. We are apologizing for the confusion.

how did you assess the patient satisfaction?

RESPONSE: Thank you for requesting clarification. Patient satisfaction was assessed verbally on a 0-100 scale. We have added “verbally on a 0-100 scale” to Lines 151.

are skin temperature sensors valid to measure the syringe temperature? mention your reference

RESPONSE: Thank you for asking for clarification. Unfortunately, we could not find any study specifically using the same skin probe for measuring syringe temperature. However, probes with the same type of sensors have been used to measure body temperature at multiple sites, including the skin (on the surface, Part#74-14902), esophagus (in space, Part#74-14905), and bladder (in liquid, Part#74-14903), all with an accuracy of ± 0.2 Celsius. The maximum heating transient time varies depending on the thickness of the probe covers, from 12 seconds for the skin sensor to 2 minutes 45 seconds for the esophageal sensor.

The sensor/probe for measuring temperature in urine (liquid) has been used clinically with FDA approval and in clinical studies. One such study published in PLOS ONE is: Cox EG, Dieperink W, Wiersema R, Doesburg F, van der Meulen IC, Paans W. Temporal artery temperature measurements versus bladder temperature in critically ill patients, a prospective observational study. PLoS One. 2020 Nov 6;15(11)

Accordingly, we have added "accuracy ± 0.2 Celsius with maximum heating transient time 12 sec" to Line 142.

3- as regard sample size, mention the used software to calculate the sample size,

the reference of the effect size, and the reference of The minimum clinically important difference (5min)

RESPONSE: Thank you for asking. The reference for the effect size and the minimum clinically important difference (5 minutes) is: Sviggum, H. P., Yacoubian, S., Liu, X., & Tsen, L. C. (2015). The effect of bupivacaine with fentanyl temperature on initiation and maintenance of labor epidural analgesia: a randomized controlled study. International Journal of Obstetric Anesthesia,

24(1), 15–21. Additionally, all anesthesia charts we have experience with include a minimum of 5-minute marks.
The sample size calculation in our department is performed using PASS: NCSS Statistical Software. The specific software version used is PASS 2016 (Power Analysis and Sample Size Software). NCSS, LLC, 2016, available at https://www.ncss.com/software/pass/.
We inadvertently missed citing this article. Therefore, we have added "(NCSS Statistical Software. PASS 2016 (Power Analysis and Sample Size Software). NCSS, LLC, 2016)" to Lines 112-113 and "3" to Line 117 for the reference mentioned earlier.

4- as regard the statistical analysis; mention the used test of normality

how did you perform the subgroup analysis in the pocket warming group to evaluate the durations of warming as reported in results

RESPONSE: On Table 2, we used the Shapiro-Wilk test for assessing normality. We apologize for the small font size and have increased it to 11 from 8 for better readability. The Shapiro-Wilk test was also used for Tables 1 (Line 198) and 3 (Line 217), and we have added this information. It is added "Shapiro-Wilk test was hired to assess normality of the data." under the section of statistical methods on Line 173.

5- in Results; in CONSORT chart, if authors performed allocation before giving the epidural, so failed procedure should be inserted in the drop-out cases that didn't receive the allocated intervention.

RESPONSE: Thank you for your comment. We have updated the CONSORT chart accordingly to reflect that failed procedures are included in the drop-out cases that did not receive the allocated intervention.

illustrate the method of the duration of pocket warming and patient satisfaction and how they were presented in results

please revise the attached file.

RESPONSE: Thank you for your illustrations that made our revision much easier. In addition to all the responses provided earlier, we have corrected the following places as specified:

1. Fig 2. Fig 2. Association of the temperature of the study medications and the lengths of their pocket warming

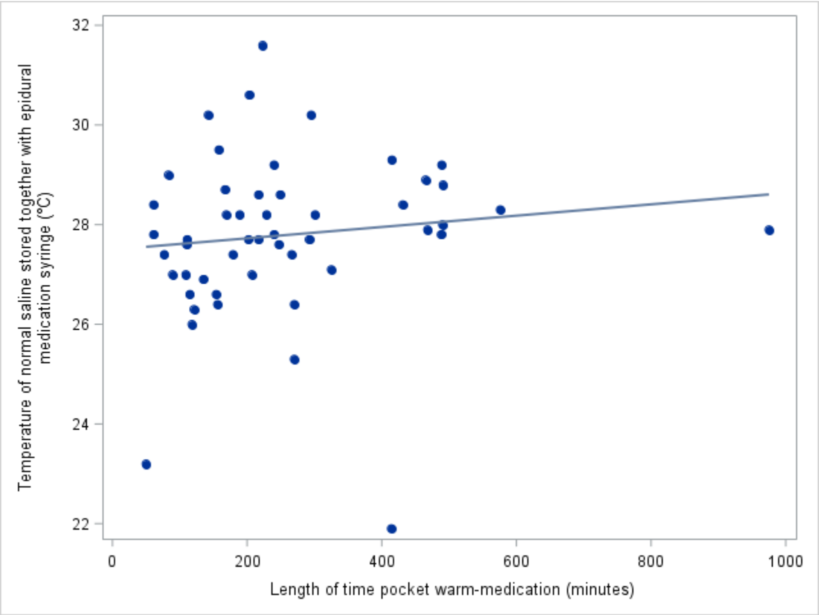

Reviewer #5: (No Response)

Reviewer #6: Thank you for giving me the opportunity to review this interesting manuscript.

This study investigated whether prewarming epidural medications in providers' pockets (to approximately 27.8°C) can shorten the onset time of labor epidural analgesia compared to room temperature medications (22.1°C). The primary outcome, median time to analgesic onset, and secondary outcomes, including incidences of adverse events such as hypotension, fever, nausea, vomiting, the number of rescue epidural boluses, and patient satisfaction rates, showed no significant difference between the groups.

It was interesting but I have some questions.

One of the most important methodological flaws of this study is the inconsistent temperature measurement of bupivacaine.

The study relied on indirect methods to estimate the temperature of the epidural solution. The temperature of the accompanying saline syringe was used to approximate the temperature of the bupivacaine solution. Direct measurement of the medication temperature immediately before administration would have provided more accurate and reliable data. This inconsistency may have influenced the study outcomes and should be addressed for greater accuracy.

Please discuss how the temperature was consistently maintained during the pocket warming and any potential variations observed.

The manuscript also does not provide detailed information specifically describing the aseptic methods used during the storage of solutions in the pocket. It is critical to ensure that proper aseptic techniques are followed to prevent contamination and infection. A detailed description of these methods should be included to strengthen the study's validity.

RESPONSE: Thank you for bringing this issue to our attention. We measured both medications using an indirect method by measuring the temperature of an accompanying 10 mL saline syringe placed next to the 10 mL syringe containing the study medication. Before our study, we found no significant temperature differences between direct and indirect measurements beyond the accuracy allowance, which was not previously described in the text. Direct measurement of the medication temperature immediately before administration is impractical since the medication needs to be administered epidurally in a sterile fashion, as you have pointed out.

To ensure proper aseptic techniques and prevent contamination and infection, we stored the medications in clean plastic bags. The syringes were double-capped, prepared by our pharmacists, and labeled with expiration dates. These details have been added to the manuscript on Lines 134-135 and Line 138 as ", double capped with labeled expiration dates," and "in clear plastic bags," respectively.

It is known that higher temperatures can cause changes in the density and viscosity of bupivacaine, which in turn affects its cephalad spread compared to cerebrospinal fluid (CSF). Studies such as Anesth Analg. 2006 Jan;102(1):272-5 have shown that a decrease in the density and viscosity of bupivacaine relative to CSF can impact its spread. This manuscript should discuss how these temperature-induced changes might influence the onset time of epidural analgesia, which is not administered intrathecally. Understanding these effects can provide insights into the observed results and help refine the study design.

RESPONSE: Thank you for providing your reference, which highlights the issues related to cerebrospinal fluid (CSF) and the effects of temperature, density, and viscosity on the onset time of bupivacaine. While these factors are relevant for intrathecal administration, the epidural space is a potential space without fluids. Therefore, the spread of medication like bupivacaine in the epidural space depends solely on injection pressure, gravity, and volume.

In clinical practice, the potential impact of temperature on the density or viscosity of bupivacaine in the epidural space has not been well studied. We could not find any studies

addressing this topic specifically unfortunately. As clinicians, we understand that the depth and level of spinal anesthesia are influenced by the density and amount of bupivacaine, while the depth of epidural anesthesia is related to the concentration of local anesthetics, and the level is related to the volume of local anesthetics.

The key difference is that intrathecal bupivacaine interacts with the existing CSF, whereas in the epidural space, bupivacaine does not mix with body fluids before affecting the nerves. Therefore, the physical characteristics of bupivacaine related to temperature, density, and viscosity may not have the same implications in epidural space as they do in intrathecal space. Due to the lack of references, limitation of our data, and limited space, we apologize that we cannot provide a more detailed discussion on this topic.

By addressing these points, the manuscript will provide a more comprehensive and accurate evaluation of the impact of prewarming bupivacaine on the onset time of labor epidural analgesia.

7. PLOS authors have the option to publish the peer review history of their article (what does this mean?). If published, this will include your full peer review and any attached files.

Reviewer #1: No

Reviewer #3: Yes: Alshaimaa Abdel Fattah Kamel

Reviewer #4: No

Reviewer #5: No

Reviewer #6: No
