# Supplementary material for: Pocket warming of bupivacaine with fentanyl to shorten onset of labor epidural analgesia: A double-blind randomized controlled clinical trial
Source: PLoS One. 2024 Sep 4;19(9):e0309515. doi: 10.1371/journal.pone.0309515 (PMC11373790; doi:10.1371/journal.pone.0309515)
Supplement: Attachment — Submitted filename: revision to authors.docx [file pone.0309515.s005.docx]

Thank you for the opportunity to review this article:

To authors,

First of all, I want to thank authors for this interesting review and for their efforts.
Title: Well written.

Abstract: well written

Background: is well written.

Methods: Thanks to authors as methods is well written in details.

Results:

- The data is statistically analyzed and expressed well.
- Tables and figures are expressed the data well.

Discussion: well written and relevant to the results of the study.

Conclusion: expressed the finding well and well written.

Best regard

Reviewer
