# Supplementary material for: Pocket warming of bupivacaine with fentanyl to shorten onset of labor epidural analgesia: A double-blind randomized controlled clinical trial
Source: PLoS One. 2024 Sep 4;19(9):e0309515. doi: 10.1371/journal.pone.0309515 (PMC11373790; doi:10.1371/journal.pone.0309515)
Supplement: Attachment — Submitted filename: Rebuttal letter for Reviewer Comments.docx [file pone.0309515.s004.docx]

Minor revisions:
1- Line 110: Include the full details for the sample size justification. The power calculation should include: (1) the estimated outcomes in each group; (2) the α (type I) error level; (3) the statistical power (or the β (type II) error level); (4) the target sample size, (5) the statistical testing method, and (6) for continuous outcomes, the standard deviation of the measurements.

*Addressed in the manuscript. “A total of 62 patients in each group would have 90% power to detect a 5-minute difference, using a one-way analysis of variance with α=0.05. These estimates were based on an effect size of 0.42 (µ_1_=4, µ_2_=9, σ=6). We intend to include 75 patients in each group (total estimated sample size of 150) to allow for patients that may not obtain adequate labor analgesia at any time after epidural placement and administration of medication. The number of patients was determined based on the sample size calculation as well as a 6.8% incidence of inadequate LEA that was previously reported at an academic center.”*

2- Line 121: If block randomization was used, state the block size.

*Addressed in the manuscript – block size of 6 was used.*

3- Line 154: Indicate the underlying covariance structure used in the mixed effects models and the criteria for selecting it.

*Addressed in the manuscript – compound symmetry was the covariance structure used in the mixed effects model. No particular pattern in the correlation among repeated measure observations was seen when evaluating the residual plots.*

4- The standard statistical term for average is mean.

*Addressed in the manuscript.*

5- Table 1: Provide only one p-value for comparing race. The one from the overall chi-square or Fisher’s exact test.

*Each category of race was captured as a separate variable and the proportions and p-values presented are the “Yes” vs “No” comparisons for each race category.*

6- Line 208: Replace the two instances of the term “stratified” because it has a specific statistical meaning in relation to the randomization process which has not been conducted in the study. Perhaps “dichotomized” is a more descriptive term.

*Addressed in the manuscript.*

7- Line 216: Provide the overall p-value for testing the interaction effect of group by time. If the interaction effect is significant, provide an interpretation of the results, but do not test main effects because the tests for main effects are uninteresting in light of significant interactions. If interaction effects are non-significant, drop the interaction effects from the model and test the main effects. Determining which results to present when testing interactions is often a multi-step process.

*Addressed in the manuscript – The overall p-value for the interaction effect of group by time was 0.418 and it was dropped from the model. Time was the only significant main effect (p<0.001).*

8- Line 223: For clarification, indicate that the secondary outcomes (rate of adverse events) did not differ significantly between the groups.

*Addressed in the manuscript.*
